# Neuroimaging of pediatric tumors of the sellar region—A review in light of the 2021 WHO classification of tumors of the central nervous system

**DOI:** 10.3389/fped.2023.1162654

**Published:** 2023-06-21

**Authors:** Rúben Maia, André Miranda, Ana Filipa Geraldo, Luísa Sampaio, Antonia Ramaglia, Domenico Tortora, Mariasavina Severino, Andrea Rossi

**Affiliations:** ^1^ Department of Neuroradiology, Centro Hospitalar Universitário São João, Porto, Portugal; ^2^ Diagnostic Neuroradiology Unit, Imaging Department, Centro Hospitalar Vila Nova de Gaia/Espinho, Vila Nova de Gaia, Portugal; ^3^ Life and Health Sciences Research Institute (ICVS), School of Medicine, University of Minho, Braga, Portugal; ^4^ Faculty of Medicine, University of Lisbon, Lisbon, Portugal; ^5^ Faculty of Medicine, University of Porto, Porto, Portugal; ^6^ Neuroradiology Unit, IRCCS Istituto Giannina Gaslini, Genoa, Italy; ^7^ Department of Health Sciences (DISSAL), University of Genoa, Genoa, Italy

**Keywords:** neuroimaging, pediatric tumors, suprasellar and sellar, CNS tumors, WHO classification

## Abstract

Sellar/suprasellar tumors comprise about 10% of all pediatric Central Nervous System (CNS) tumors and include a wide variety of entities, with different cellular origins and distinctive histological and radiological findings, demanding customized neuroimaging protocols for appropriate diagnosis and management. The 5th edition of the World Health Organization (WHO) classification of CNS tumors unprecedently incorporated both histologic and molecular alterations into a common diagnostic framework, with a great impact in tumor classification and grading. Based on the current understanding of the clinical, molecular, and morphological features of CNS neoplasms, there have been additions of new tumor types and modifications of existing ones in the latest WHO tumor classification. In the specific case of sellar/suprasellar tumors, changes include for example separation of adamantinomatous and papillary craniopharyngiomas, now classified as distinct tumor types. Nevertheless, although the current molecular landscape is the fundamental driving force to the new WHO CNS tumor classification, the imaging profile of sellar/suprasellar tumors remains largely unexplored, particularly in the pediatric population. In this review, we aim to provide an essential pathological update to better understand the way sellar/suprasellar tumors are currently classified, with a focus on the pediatric population. Furthermore, we intend to present the neuroimaging features that may assist in the differential diagnosis, surgical planning, adjuvant/neoadjuvant therapy, and follow-up of this group of tumors in children.

## 1. Introduction

Central nervous system (CNS) tumors constitute the most common type of solid tumor of childhood, representing approximately 27% of pediatric cancers, closely surpassed by leukemia (28%). On the other hand, CNS tumors in adolescents are slightly more frequent than leukemia, comprising 21% of all cancers (1). Overall, more than 4,000 CNS tumors are diagnosed each year in the pediatric age group (2), accounting for most cancer-related deaths in this population (3).

Sellar and suprasellar tumors comprise about 10% of all pediatric CNS tumors and include a wide range of different entities, covering various histologic origins and radiological findings, in many instances presenting distinct clinical and neuroimaging characteristics and demanding individualized surgical approaches and/or medical treatments (4).

After incorporating the definition of new entities based on molecular characteristics for the first time in the World Health Organization (WHO) Revised 4th Edition Classification (5), the most recent “Blue Book”—the fifth edition of the WHO Classification of Tumors of the Central Nervous System (WHO CNS5) -, (6) kept building on the previous one, in line with the rapidly evolving molecular updates, and unprecedently incorporated both histologic and genetic alterations into a common diagnostic framework while making relevant changes in the way CNS tumors are classified and graded (7). Importantly, it is now acknowledged that childhood tumors are fundamentally different from those occurring in adults, as can be noticed by the separation of diffuse gliomas in adult and pediatric types. Some age-relevant changes also occurred in the classification of tumors of the sellar region: adamantinomatous (ACP) and papillary craniopharyngiomas (PCP), for example, were previously considered two sub-types of craniopharyngioma but are now classified as two separate tumor types given their several differences, including demographic distribution. Also pituitary blastoma is a newly added embryonal tumor of infancy (6).

Neuroimaging plays a critical role in the detection, diagnosis, treatment planning and follow-up of sellar and suprasellar tumors. However, imaging features are only briefly mentioned in the WHO CNS5, and even less when it comes to pediatric population specificities.

In this review we intend to fill the gap between the recent genetic and molecular advances that gave rise to the recently published WHO CNS5 and the neuroimaging features that may assist in differential diagnosis, surgical planning, adjuvant therapy, and follow-up of sellar and suprasellar tumors in pediatric patients (Table 1).

**Table 1:** Pediatric tumors of the central nervous system with possible sellar localization, as classified by the WHO CNS5.

Gliomas, glioneuronal tumours, and neuronal tumours
*Circumscribed astrocytic gliomas*
Pilocytic astrocytoma
Embryonal tumours
*Other CNS embryonal tumours*
Atypical teratoid/rhabdoid tumour
Haematolymphoid tumours involving the CNS
*Histiocytic tumours*
Juvenile xanthogranuloma
Langerhans cell histiocytosis
Germ cell tumours
Germ cell tumours of the CNS
Tumours of the sellar region
Adamantinomatous craniopharyngioma
Papillary craniopharyngioma
Pituicytoma, granular cell tumour of the sellar region, and spindle cell oncocytoma
Pituitary adenoma/pituitary neuroendocrine tumour
Pituitary blastoma
Genetic tumour syndromes involving the CNS
Neurofibromatosis type 1
*DICER1* syndrome

## 2. Anatomy, embryology, and normal imaging features

The pivotal structure of the sellar region is the pituitary gland, or hypophysis. It is an ovoid body continuous with the infundibulum, or pituitary stalk, and lies within the sella turcica where it is confined superiorly by a diaphragm of dura mater. Both the posterior neurohypophysis and the anterior adenohypophysis include part of the infundibulum, although the former is a diencephalic downward extension connected to the hypothalamus through the infundibular stem, and the latter an oral ectodermal derivative resulting from an upgrowth invagination of the stomodeum roof—the Rathke's pouch.

Both down- and upgrowth processes are present by the third gestational week. The Rathke's pouch disconnects from the oral cavity at about 32 days of gestation and, 10 to 12 days later, it originates the anterior pituitary lobe. The anterior wall of the pouch will form the *pars distalis* while the posterior wall will give rise to the *pars intermedia*. As this growth continues, the pouch progressively narrows its lumen becoming a cleft that is usually not recognizable in the adult pituitary gland, despite being a potential site for cysts. The portion of the Rathke's pouch extending from the skull base to the floor of the sella turcica eventually disappears, but nests of pituitary tissue can sometimes persist along its course. At the same time, the neurohypophysis originates from the neuroectodermal neurohypophyseal diverticulum and infundibulum, giving rise to the median eminence, infundibular stem and posterior lobe; neuroepithelial cells proliferate at the distal part of the infundibulum, later differentiating into pituicytes, the primary cells of the posterior lobe.

The adenohypophysis makes up about 75% of the gland and is divided in three parts: the *pars distalis*, the *pars intermedia,* and the *pars tuberalis.* The *pars distalis* is the larger and most anterior part, separated dorsally from the *pars intermedia* by a fetal and early postnatal hypophyseal cleft, a vestige of Rathke's pouch which may persist as a cystic cavitation, and continuous with *pars tuberalis,* which encircles the neurohypophyseal axons in the infundibular stem. A recently discovered septation was described to be positioned behind the *pars intermedia* and *pars tuberalis*, fused superiorly with the arachnoidal trabeculus under the optic chiasm, separating the adeno- from neurohypophysis (8). The neurohypophysis includes the posterior lobe proper, the infundibular stem and the median eminence.

The hormonal supply of the neurohypophysis occurs through a direct fiber continuity with the hypothalamus, that releases oxytocin- and vasopressin-containing vesicles in the posterior lobe. On the other hand, the adenohypophysis does not have axonal continuity with the hypothalamus, relying instead on a hypothalamic-hypophyseal portal system which provides a means of transportation of parvocellular messengers to the anterior gland. Adenohypophyseal hormones include follicle stimulating hormone, luteinizing hormone, corticotropin, thyroid-stimulating hormone, prolactin, and growth hormone.

On MRI, the fetal and neonatal (term and preterm) adenohypophysis shows a characteristic high T1 signal, which decreases linearly after birth and becomes isointense with the pons by 6–8 weeks of life. This process has shown to be independent of the gestational age at birth, proving to be part of extrauterine life adjustments (9, 10). Typical T1 hyperintensity of the neurohypophysis (the so-called “bright spot”) becomes apparent (due to presence of the so-called vasopressin-neurophysin II-copeptin complex, responsible for the vasopressin storage) and is a marker of integrity of the hypothalamic-hypophyseal tract (10, 11). Intense post-gadolinium enhancement of the adenohypophysis and of the infundibulo-tuberal region is well evident at all ages due to absence of blood-brain barrier (12).

Even after correcting for gestational age, preterm infants have taller glands than full-term infants, which is thought to result from reduced insulin-like growth factor 1 and higher levels of growth hormone in premature infants (13). At birth, the pituitary gland has an upward convex margin in both pre- and full-terms, and its height remains stable or slightly decreases during the first 2 years of life. In prepubertal children, normal pituitary gland height values range between 3 and 6 mm. Then, a slight but progressive increase in height takes place until puberty, where it can reach 10–11 mm in girls and 7–8 mm in boys (12). Useful reference tables with normal pituitary gland and stalk morphometric parameters according to age specifically for children are available (14).

**Figure 1:**
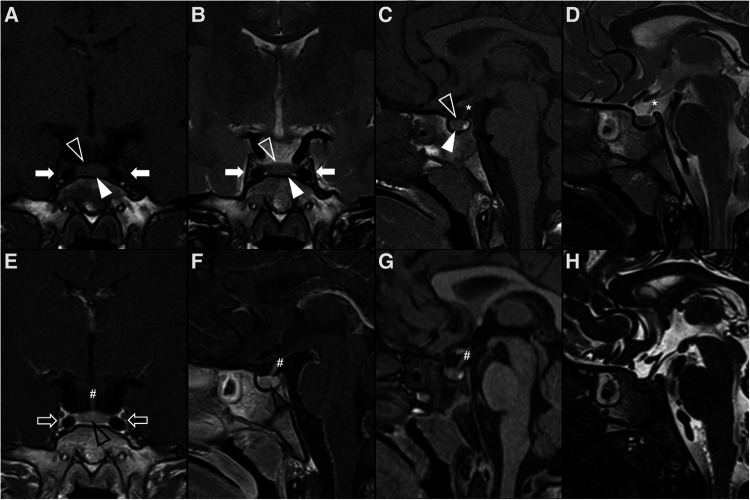
Normal appearing pituitary gland and sella in a 6-year-old girl. Brain MRI including coronal (**A**) and sagittal (**C**) T1WI as well as coronal (**B**) and sagittal (**D**) T2WI reveal a normal sized sella turcica as well as regular volume and morphology of the adenohypophysis according to age and gender (white arrowhead). Note symmetry and homogeneous signal intensity of the gland and flattened superior border (empty white arrowhead). The pituitary stalk is in the midline and presents regular thickness (asterisks). The spontaneous T1 hyperintensity of the neurohypophysis, related to vasopressin storage, is better depicted in sagittal imaging in the posterior aspect of the sella turcica (black arrowhead). Also note normal permeability of the suprasellar cistern and regular lateral concavity of the cavernous sinuses (white arrows). Coronal (**E**) and sagittal (**F**) T1WI after gadolinium injection reveal normal homogeneous enhancement of the pituitary gland (empty black arrowhead), pituitary stalk (hashtag), and cavernous sinus (empty white arrows) due to absence of blood brain barrier. MR imaging should also include pre (**G**) and post-contrast volumetric T1 MPRAGE imaging not shown) for morphological characterization of brain as well as better depiction of focal areas of hypo-enhancement within the pituitary gland or characterization of other sellar/suprasellar masses, respectively. Sagittal 3D T2-weighted sequences (e.g., CISS) (**H**) a**r**e useful in the anatomical characterization of the sellar region including pituitary gland as well as other midline structures and cystic lesions.

Physiological magnetic resonance imaging (MRI) features are depicted in Figure 1 and expected variations along the several stages of development are shown in Supplementary Figure S1.

## 3. Imaging technique and suggested protocol

Imaging of the sellar region has greatly evolved over the years. In a historical perspective, plain radiography, pneumoencephalography and angiography were once imaging modalities with very limited but real value in the indirect detection of sellar/suprasellar masses. Computed tomography (CT), with its cross-sectional properties, revolutionized intracranial imaging, but magnetic resonance imaging (MRI) is nowadays the main imaging modality to assess sellar and suprasellar regions at all ages because of its superior tissue contrast resolution; moreover, it is particularly useful in children since it does not employ ionizing radiation. However, low dose CT scan remains a valuable alternative to MRI when the latter is unavailable or contraindicated, and may provide complementary information for diagnosis and surgical planning. In particular, it allows assessment of soft tissue calcifications and hemorrhage as well as bony destruction, hyperostosis or remodeling. In addition, when considering a transsphenoidal surgical approach, it is important to identify the degree of sphenoid sinus pneumatization and the intercarotid distance (15).

Dedicated sellar region MRI in the pediatric population is usually performed following clinical and/or laboratory neuroendocrine findings including precocious puberty, hypopituitarism or central diabetes insipidus, and less frequently, hyperprolactinemia or Cushing's syndrome.

When imaging the pituitary region, the most relevant information is provided by high-resolution spin-echo/turbo spin-echo T1-weighted imaging (WI) and T2WI obtained on sagittal and coronal planes, with 2–3 mm slices and a small field of view, and preferably using a 3 T equipment (16). Whole brain volumetric gradient-echo T1 sequences can be also acquired as an alternative or a complement to spin-echo T1W images of the sellar/suprasellar region.

Sagittal planes allow the visualization of the pituitary stalk, the infundibulum and the optic chiasm, as well as the assessment of the intrinsic T1 hyperintensity of the posterior pituitary (17). On the other hand, the borders between the sellar compartment and the parasellar/cavernous sinuses structures are better evaluated on coronal T2W images. Furthermore, both coronal and sagittal planes provide important information about the position of the stalk related to the midline.

Although congenital abnormalities and isolated growth hormone defects usually do not require contrast administration to be characterized, it is widely accepted that gadolinium-based contrast adds additional benefit in identifying otherwise occult small pituitary lesions (as it may be the case with microadenomas) as well as in the characterization of sellar/suprasellar masses and/or detection of eventual leptomeningeal or ependymal dissemination (12, 17). In particular, dynamic contrast-enhanced T1WI coronal images are used to increase the sensitivity detecting small adenomas within the pituitary as these lesions show delayed enhancement comparing with the normal gland parenchyma. This sequence consists in the injection of a rapid intravenous bolus of paramagnetic contrast at a dose of 0.1 ml/kg followed by sequential imaging every 10–12 s for 60–90 s, 1–2 min after contrast administration (18, 19). More recently, 3D gradient-echo T1WI (20), 3D T2W SPACE (21), and Fluid Attenuated Inversion Recovery (FLAIR) (22) sequences after gadolinium administration proved to further improve the detection of ACTH-secreting microadenomas.

We also believe that 3D heavily T2W images (FIESTA/DRIVE/CISS) acquired in the sagittal plane are useful to evaluate the sellar/suprasellar region, namely the pituitary stalk thickness and other midline structures, including the median eminence, tuber cinereum, mammillary bodies, pineal gland or Liliquist membrane, which may hardly be delineated otherwise (12). This sequence is particularly relevant in case of suprasellar masses, in order to assess the degree of tumoral invasion and/or loco-regional mass effect.

In spite of the hypothalamic-pituitary system being the main object of study in the above-mentioned clinical settings, the necessity for additional whole intracranial assessment should be considered on an individual basis. It is advisable to rule out additional brain abnormalities and/or lesions by acquiring axial FLAIR or T2WI (depending on the age of the child) and axial Diffusion Weighted Imaging (DWI) sequences as well as additional sequences tailored according to the clinical suspicion and imaging findings.

Moreover, it is very important to mention that whenever a mass lesion with the potential of secondary dissemination is identified in the sellar/suprasellar region the whole neuroaxis should be imaged (12), and angiographic studies should be also considered for evaluation of the degree of encasement of the adjacent vessels.

A suggestion of sellar/suprasellar MR protocol based on the authors' experience is listed in Table 2.

**Table 2:** Recommended sellar and parasellar MR protocol in children.

Sequence	Plane	Mode	Thickness	Estimated time (3.0 Tesla scanner)
T1 TSE	Coronal and Sagittal	2D	2–3 mm	17 min
T2 TSE	Coronal and Sagittal	2D	2–3 mm	
T1 TSE C+^*^^#^	Coronal and Sagittal	2D	2–3 mm	
**Optional**				
T1 MPRAGE	Sagittal	3D	0.6–1 mm	3.5 min
T2 DRIVE	Sagittal	3D	0.6–1 mm	4.5 min
T1 TSE DCE	Coronal	2D	2–3 mm	2 min^§^

If a sellar/suprasellar mass is identified, brain MRI ± time-of-flight MR angiography and whole spine MRI should be added to the protocol.

^*^ 0.1 mmol/kg dose injection with a gadolinium chelated contrast agent. Use of a power injector is desirable at an injection rate of 3–5 cc/s.

^#^ Sequence acquisition should be performed 5-minute post-contrast injection.

^§^ Dynamic study should not exceed 20 s per slice.

For neonatal and early infancy MRI studies, a feed-and-wrap/swaddle technique should be attempted at least once, although it may require adjustments to the protocol (23). For older uncooperative children (usually between 6 months to five years), sedation/general anesthesia is often required, especially when evaluation of the whole neuroaxis is necessary. Nevertheless, their usage should be avoided whenever possible through diverse available techniques, including specific preparation, distraction strategies, as well as motion correction during or after image acquisition (23).

## 4. Sellar and suprasellar tumors

### 4.1. Adamantinomatous craniopharyngioma

Adamantinomatous craniopharyngioma is a WHO grade 1 mixed solid and cystic squamous epithelial tumor with stellate reticulum and wet keratin and characterized by activating mutations on *CTNNB1* (6). This gene encodes the WNT signaling pathway regulator β-catenin (24) and is identified in as many as 100% of ACP samples when using the most sensitive sequencing methods (25). It has been postulated that these tumors arise from ectoblastic remnants of the Rathke's duct and therefore are commonly found along the path of development of the Rathke's pouch from the pharynx to the hypothalamus crossing the sellar and suprasellar region, most commonly along the hypothalamic-pituitary axis (26).

ACP account for almost all craniopharyngiomas in children and also for 5%–11% of non-glial intracranial neoplasms in this age group (6). In turn, ACP represent approximately 80% of adult craniopharyngiomas. Congenital cases are rare but have been reported (27). No sex predilection has been described (28).

Craniopharyngiomas may be rarely detected incidentally (29). Affected children usually present features of raised intracranial pressure and endocrine dysfunction including delayed puberty and short stature. Visual impairment may be also present in up to 50% of pediatric cases and is the most common change in adults (30, 31). Hormonal deficits may also occur at all ages, but are less pronounced in childhood-onset craniopharyngioma when compared to adults (32). Such deficits include hypothalamic dysfunction such as diabetes insipidus (33). A clinical picture of headache, visual impairment, decreased growth rate, and polydipsia/polyuria should arise suspicion of childhood craniopharyngioma (34). They often occur in the sellar and infundibulotuberal region, with around 95% having a suprasellar component. Indeed, ACP usually have both intrasellar and suprasellar components (53%–75%), followed by purely suprasellar (20%–41%), and least commonly purely intrasellar (5%–6%) (35) They are partly solid and cystic and can show compact areas of calcification. The fluid within the cysts is classically characterized as “machine oil”-like. The microscopic appearance is of an external layer of columnar epithelium and a central network of epithelial cells, with keratin deposits in the cellular stroma (31). These lobulated masses have nonuniform surfaces that strongly adhere to surrounding gliotic parenchyma *via* finger-like tumor protrusions with numerous Rosenthal fibers (6).

**Figure 2:**
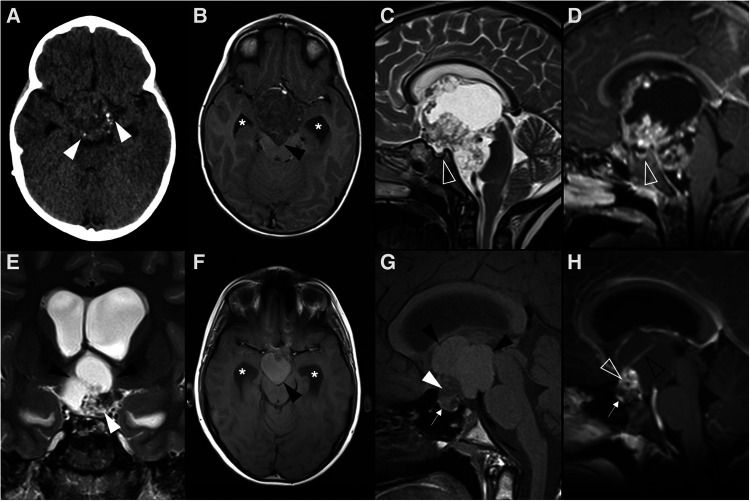
Adamantinomatous craniopharyngioma in 6-yearl-old boy (**A–D**) presenting with headaches. Axial head CT scan (**A**) shows an iso-hypodense expansile suprasellar lesion with multiple foci of calcification in the periphery (white arrowheads). Brain MR including axial T1WI (**B**), sagittal T2WI (**C**) and sagittal T1WI after gadolinium injection (**D**) shows a very large expansile lesion with heterogeneous signal intensity and enhancement, causing obliteration of the III ventricle and presenting extension towards the interpeduncular (black arrowhead) and pre-pontine cisterns with associated mass effect over the optic chiasm and optic tracts as well as brainstem. Note normal positioning and enhancement of the pituitary gland (empty white arrowhead) as well as moderate hydrocephalus (*). Adamantinomatous craniopharyngioma in a 13-year-old (**E–H**) boy presenting with headaches. Brain MRI including coronal T2WI (**E**) as well as axial (**F**) and sagittal (**G**) T1WI reveal a multilobular lesion centered in the suprasellar cistern and extending towards the interpeduncular cistern, separated from the pituitary gland, showing heterogenous signal intensity, including an inferior solid component (white arrowheads) and multiple cysts with spontaneous hyperintensity on T1 and T2 (black arrowheads). Post-contrast sagittal T1WI (**H**) reveals nodular enhancement of the solid component (empty white arrowhead) as well as peripheral enhancement of the cysts (empty black arrowhead). There is associated mass effect over the III ventricle, optic chiasm and tracts as well as moderate hydrocephalus (*).

The imaging features of ACP are quite characteristic and have been condensed by Castillo and Mukherji (36) as the “90% rule”: “90% are cystic, 90% have calcification, 90% enhance, and over 90% have suprasellar component” (Figure 2). Therefore, a multilobulated, multicystic, contrast-enhancing suprasellar lesion with calcification, is almost certainly a craniopharyngioma (10). The cystic components predominate in children and may contain highly proteinaceous fluid, accounting for the non-suppressed signal in FLAIR sequences and for the T1W hyperintensity frequently seen when the protein concentration equals or surpasses 90 g/L, although T1W hypo- or isointensity can also be noticed (37). The cyst wall enhances after gadolinium administration and is more commonly thin with variable nodularities included. The solid components are iso- to hyperintense in T1WI, have variable signal intensity in T2W images, partially as a result of calcification, and usually enhance after paramagnetic contrast is given (38). Calcification is a hallmark of ACP but sometimes challenging to depict in MRI, thus non-enhanced CT scans should always be performed. They can appear as shell-like deposits along the cyst wall or form punctate or bulky areas within the lesion (12). Both calcifications and hydrocephalus are more frequent in children than in adults (39). Proton MR spectroscopy of pediatric craniopharyngiomas has distinct characteristics from other suprasellar tumors, showing no normal metabolites and a dominant peak at 1 to 2 ppm, consistent with lactate or lipids (40).

Various degrees of deformation of the ventricular system can be seen, most often by elevation of the floor of the third ventricle, a finding that positively correlates with poor hypothalamic function (41). Moreover, preoperative hyperphagia and higher body mass index are also associated to some MRI features, namely unidentifiable pituitary stalk, chiasm displacement and hypothalamic edema with elevated signal in T2W/FLAIR sequences (39). Interestingly, the extent of preoperative hypothalamic involvement on imaging was found to be the only independent risk factor for postoperative weight changes, namely hypothalamic obesity, which can be seen in around 50% of pediatric cases (39, 42). A complete definition of tumor anatomic interplay on the hypothalamus in preoperative MRI is of paramount importance to set a resection goal (39).

Given the clinical importance of hypothalamic damage after surgery, de Vile et al. (43) developed a three-tiered classification system based on neuroimaging criteria, showing an association between the rate of severe obesity and the grade of hypothalamic damage, confirmed by several subsequently published extended classifications (44).

Radical resection is associated with better outcomes and is therefore considered the primary therapy of choice. However, the rate of firm hypothalamic adherence is up to 26.8% of cases, accounting for variable extent of resections (39) and claims of non-superiority when compared with subtotal resection followed by adjuvant radiation (6). Surgical sparing of the posterior hypothalamus is associated with higher quality of life and decreased development of obesity, but it comes with lower progression-free survival (45). Local infiltration of the hypothalamus, visual tract, and arteries of the circle of Willis is seen in approximately 25% of surgical specimens. Subarachnoid dissemination or implantation along the spinal cord, the surgical track, or the path of needle aspiration is very rare (6).

Although classified as a benign (WHO grade I) tumor, a good prognosis is often hampered by invasion of adjacent structures, precluding safe gross total resection. Using a multivariate model, the systematic definition of three preoperative MRI features has proven valuable to predict more severe tumor adherence to the hypothalamus: the position of the hypothalamus around the mid-third portion of the tumor, signs of amputated or infiltrated stalk, and multilobulated and dumbbell tumoral shapes (46).

Malignant transformation of craniopharyngiomas is an exceedingly rare phenomenon which, however, mostly occur in the adamantinomatous type, especially during childhood (47). Overall survival rates that have been described in pediatric series are 83%–96% at 5 years, 65%–100% at 10 years, and 62% at 20 years (6). Recurrence and regrowth of pediatric craniopharyngiomas are seen in 17%–36% following gross total resections, and are usually diagnosed in asymptomatic patients in a routine follow-up MRI (48, 49).

### 4.2. Papillary craniopharyngioma

The WHO CNS5 definition of PCP is of a grade 1 solid or partially cystic, non-keratinizing squamous epithelial tumor that develops in the infundibulotuberal region of the third ventricle floor, most often in adults. This tumor is characterized by *BRAF* p.V600E mutations, that are present in almost all cases, leading to activation of the MAPK/ERK pathway (6). Therefore, despite originating from similar stem cell populations, PCP and ACP have distinct methylation and transcriptional profiles (50).

Overall, PCP account for about 10% of all craniopharyngiomas and 10%–33% of those arising in adults, occurring mainly in males. One should keep in mind that PCP are rare among young patients and the majority of pediatric cases were reported in individuals between 17 and 18 years (51, 52). In a recent study, among 226 craniopharyngiomas analyzed with regard to growth patterns (53), in fact no PCP were found before the age of 17.

Due to their rarity during childhood, imaging features of PCP in published literature tend to overlook pediatric specificities. Previous reports have suggested that a suprasellar/supradiaphragmatic location is a strong predictor of *BRAF*-mutated craniopharyngiomas (54–56). However, in children these lesions usually arise in the sellar region, expanding the pituitary fossa and growing into the suprasellar region, therefore acquiring a round or elliptic shape (57–60). Pediatric PCP also seem to differ from their adult counterparts by consistently showing prominent cystic components with enhancing walls (60). In contrast to ACP, no egg-shell calcifications are found in both adult and pediatric PCP, although scattered high-density intratumoral substances are frequently seen (60). Therefore, the imaging appearance of pediatric PCP can be undistinguishable from Rathke's cleft cysts (RCC), which are also rounded and intrasellar cystic lesions that can enlarge to the suprasellar region in up to 87% (4) and may show enhancing margins corresponding to the compressed pituitary (12). Furthermore, squamous metaplasia is observed in 5%–17% of RCC cases, extending the diagnosis challenge to the field of histology (61). However, RCC do not harbor *BRAF* mutations.

Despite its rarity in the pediatric population, considering PCP as a possible diagnosis can strongly influence patient management if the tumor cannot be completely resected at once, as targeted therapy for *BRAF* gene can achieve tumor volume reduction.

Because of the low prevalence of PCP among all pediatric craniopharyngiomas, information about prognosis is very limited. However, it is generally accepted that PCP have a better prognosis than ACP due to being less adherent to adjacent structures and therefore easier to resect (62).

### 4.3. Posterior pituitary tumors: pituicytoma, granular cell tumor of the sellar region, and spindle cell oncocytoma

Pituicytoma, granular cell tumor of the sellar region, and spindle cell oncocytoma (SCO) are all posterior pituitary tumors and have been agglutinated in the same category, as they constitute a distinct group of low-grade neoplasms showing expression of thyroid transcription factor 1 (TTF1). In addition, all arise from pituicytes of the posterior pituitary (6), that in turn are GFAP immunoreactive spindle or stellate cells believed to regulate neurohypophyseal hormone secretion (63).

Pituicytoma was first characterized in a series published in 2000 (64) and then became an official WHO entity. The range of different morphologies seen in histologic studies is supported by the existence of five subtypes of pituicytes within the neurohypophysis, including oncocytic, granular and “major” (astrocyte-reminiscent) pituicytes (65). The ultrastructural variants of pituicytes are reflected in the 3 morphologic variants of tumors arising from these cells.

Several historical synonyms for this group of tumors have been used in the literature and are currently not recommended: choristoma, granular cell myoblastoma, infundibuloma, pilocytic astrocytoma, and granular cell tumor are now discouraged when referring to pituicytoma; similarly, Abrikossoff tumor and spindle cell oncocytoma of the adenohypophysis are no longer supported designations for granular cell tumors of the sellar regions and SCO, respectively (6).

Posterior pituitary tumors are rare and not usually suspected by clinicians in the differential diagnosis of the sellar masses. Furthermore, clinical presentation is not specific, including headaches, visual field defects, and hypopituitarism, and they usually have hormonal profile and radiological findings resembling non-functioning pituitary adenoma. The inability to distinguish these lesions is relevant because they tend to be more vascular and prone to heavy bleeding during surgical resection (66–68).

A recent meta-analysis identified about 270 cases of the entire group, where only 7 cases were pediatric ones (69). Thus, there is scarce neuroimaging information available to characterize these tumors in the pediatric population.

**Figure 3:**
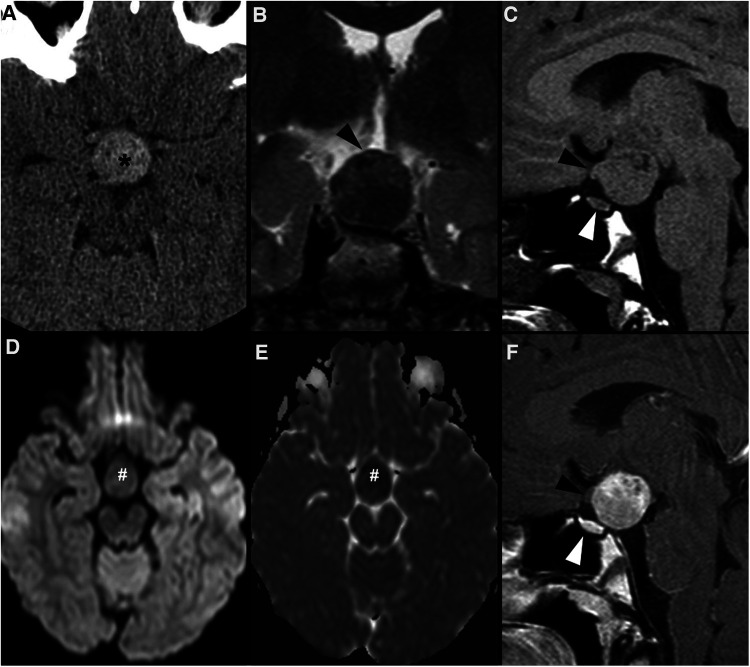
Granular cell tumor of the neurohypophysis in a 12-year-old boy with visual disturbances and central diabetes insipidus. Imaging shows a compact, solid and rounded suprasellar lesion, spontaneously hyperdense (asterisks) on axial head CT scan (**A**). Brain MRI shows that the mass is well-defined and hypointense on coronal T2WI (**B**) and isointense on sagittal T1WI (**C**) (black arrowheads). There is mild reduced diffusivity on axial DWI (**D**) and ADC map (**E**), and homogenous solid enhancement on post-gadolinium sagittal T1WI (**F**). The expansile lesion causes superior and anterior displacement of the optic chiasm (black arrowheads in **C,F**) and deformation of the floor of the third ventricle. Note normal location and enhancement of the pituitary gland on sagittal images [(**C,F**) white arrowhead].

Imaging features of 112 posterior pituitary tumors were reviewed in a meta-analysis and included 64 granular cell tumors, 35 pituicytomas, and 13 SCO (70). Most lesions were both intrasellar and suprasellar masses despite pituicytoma and granular cell tumor being the only ones ever presenting as purely intrasellar and suprasellar lesions, respectively. No SCO were exclusively intra- or suprasellar and, in addition, they have the tendency to extend to the cavernous sinus and invade the sellar floor (71). Three quarters of all tumors were infiltrating and could not be separated from the underlying pituitary gland. Almost all tumors showed intermediate T1 signal, while pituicytomas were generally hyperintense on T2WI compared with gray matter, and granular cell tumors were predominately isointense. Among tumors of posterior pituitary, pituicytoma was the only one to show a more predictable homogeneous enhancement pattern. Magnetic resonance images of a case of granular cell tumor of the neurohypophysis are shown in Figure 3.

### 4.4. Pituitary adenoma/pituitary neuroendocrine tumors

The new WHO classification features a major nomenclature change, namely substituting the traditional term “adenoma” for the more accurate name “pituitary neuroendocrine tumor” (PitNET) since the hormone-secreting cells of the adenohypophysis are neuroendocrine cells. These tumors have been classified as adenomas based on the rarity of metastatic behavior, ignoring that the term also implies a benign behavior and a rather harmless disease course, which is not the case for a considerable number of these tumors. Indeed, tumors of the adenohypophysis are often invasive neoplasms that can infiltrate into surrounding structures and even metastasize, while there is no such thing as metastasizing adenomas. Therefore, the term “metastatic PitNET” is now advocated to replace the previous “pituitary carcinoma” in order to avoid confusion with neuroendocrine carcinoma (72).

Although this class of tumors has been classically categorized into micro- or macro-lesions according to their size, more specifically if they are smaller or larger than 10 mm (12), these descriptive terms are not part of the WHO CNS5 classification. Nevertheless, for description purposes, we will maintain their distinction in this review.

PitNETs are a clonal neoplastic proliferation of anterior pituitary hormone–producing cells, mostly occurring in a sporadic way, although ∼5% of all cases present in a familial setting and over half of these are due to multiple endocrine neoplasia type 1 (MEN-1) and Carney's complex disorders (73, 74). PitNETs are also associated with rare phakomatoses, such as the McCune-Albright and Nelson syndromes (75, 76).

These tumors represent 2%–8.5% of pituitary tumors in patients under the age of 20 but make up less than 3% of the supratentorial tumors in children, therefore being relatively uncommon during childhood (15, 77). During the pediatric age, they are more frequent in adolescents than in younger age groups, and while in the former they are more often seen in girls than boys, it is noteworthy that no gender predilection is found among pre-pubertal patients (78). In contrast to adult counterparts, the vast majority (about 90% of cases) of pediatric PitNETs requiring surgery are mainly prolactin- or ACTH-producing tumors (79, 80).

Clinical manifestations are variable according to age and gender as well as size and secreting status of the lesion. Most prolactin-secreting PitNETs occur in girls (5:1) and will present during or after puberty, with primary or secondary amenorrhea due to excessive prolactin secretion (81). On the other hand, the chief complaints in pediatric ACTH-secreting PitNET include rapid and substantial weight gain as well as growth failure (82). Somatotropin-secreting tumors are mainly found in boys and may manifest as gigantism or acromegaly, depending on whether the presentation occurs before or after the fusion of long bone growth plates, respectively (79). Thyrotropin-secreting and non-functioning PitNETs are rarely diagnosed before adult age and tend to manifest with symptoms of mass effect, although visual defects are less frequent than in adults. Pituitary apoplexy seldom occurs in children, and hypopituitarism is less likely to follow (15, 83).

**Figure 4:**
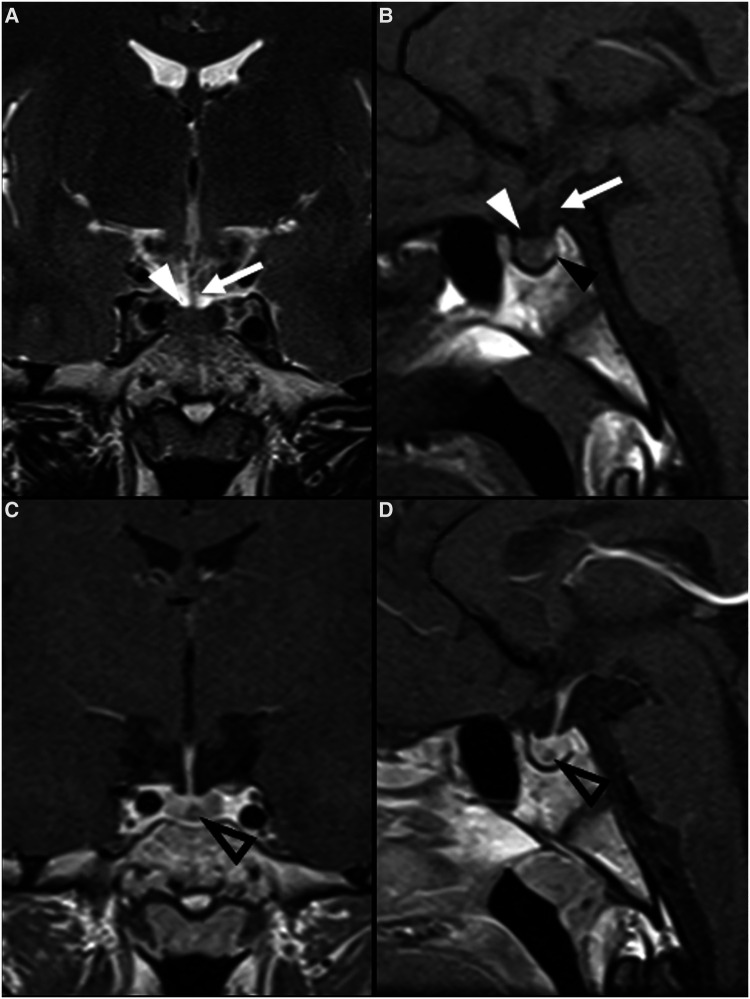
Pituitary neuroendocrine tumor in a 15-year-old boy with ACTH-dependent cushing syndrome. Coronal T2WI (**A**) and sagittal (**B**) T1WI reveal a normally sized sella turcica as well as a pituitary gland with age and gender appropriate volume and preserved superior concavity (white arrowhead). There is also adequate positioning and thickness of the pituitary stalk (long arrow) and normal spontaneous T1 hyperintensity of the neurohypophysis (black arrowhead). Coronal (**C**) and sagittal (**D**) T1WI after gadolinium injection reveal a small, rounded and midline non-enhancing lesion within the adenohypophysis with a diameter smaller than 1 cm (empty white arrowhead**s**) as well as normal enhancement of the remainder glandular parenchyma.

The imaging features of pediatric PitNET do not differ considerably from those of adults. Similar to older patients, PitNETs smaller than 10 mm (previously microadenomas) may be difficult to visualize or may be indistinguishable from RCC or *pars intermedia* cysts, favoring the implementation of MRI protocols centered on the pituitary gland (Figure 4). They appear as small T1 hypointense or isointense masses within the adenohypophysis, with variable intensity in T2WI. On thin section coronal dynamic contrast-enhanced imaging (DCEI), these lesions show relative low signal intensity comparing to the rapidly enhancing pituitary gland during early image acquisition, progressing to isointensity at later time points, due to their delayed contrast enhancement (84). ACTH-producing PitNETs are known to be particularly challenging to depict on neuroimaging studies due to their typically small size and showing a similar pattern of enhancement to the surrounding gland (85); it has been reported that post-gadolinium gradient echo 3D T1 sequences are more sensitive than DCEI in identifying this subtype of lesions (86). Other imaging features such as deviation of the infundibulum and asymmetry of the gland or the sellar floor, if present, will help to identify the lesion (15).

**Figure 5:**
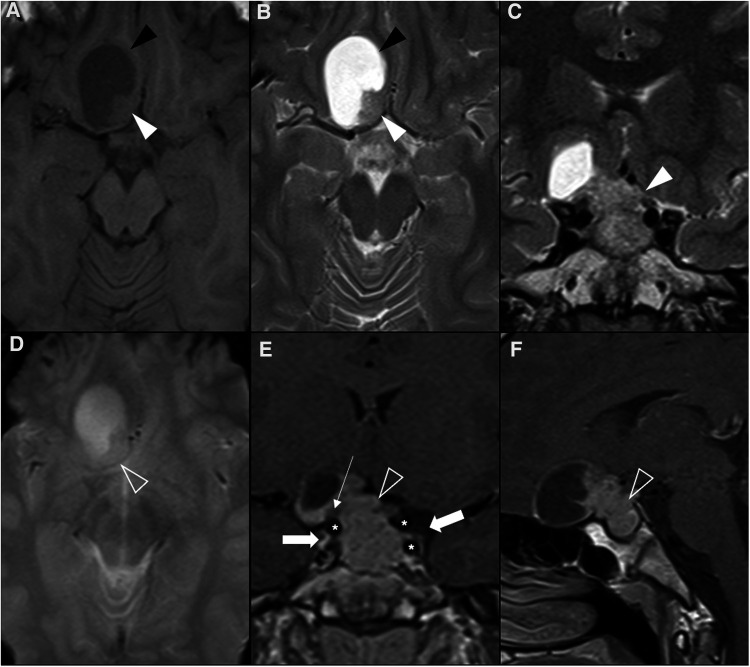
Prolactin-releasing pituitary neuroendocrine tumor in a 16-year-old boy presenting with headaches. Brain MRI including axial (**A**) T1WI as well as axial (**B**) and coronal (**C**) T2WI shows an expansile sellar and suprasellar lesion resembling a “snowman” appearance that cannot be differentiated from the pituitary gland, mainly isointense in T1WI and iso-hyperintense on T2WI (white arrowhead), and with a round hypointense T1 and hyperintense T2 cystic component (black arrowhead) located in the right anterior region and causing displacement of the right rectus and orbital gyri without associated edema. No associated paramagnetic foci suggestive of calcifications are depicted on axial (**E**) T2 gradient-echo imaging. Coronal (**F**) and sagittal (**G**) T1WI after gadolinium injection reveal homogenous enhancement of the solid component of the lesion (empty white arrowhead). There are no signs of cavernous sinus invasion, more prominent on the right (white arrows), without stenosis of the cavernous segments of the internal carotid arteries (asterisks). Note that the pre-chiasmatic segment of the right optic nerve is encased by the lesion [long white arrow in (**F**)], with significant adherence confirmed intraoperatively. Carotid arteries are marked with asterisks in (**F**).

When larger than 10 mm, PitNETs consist of overt lesions that fill the sella, frequently expand upwards to the suprasellar region, and grow laterally towards the cavernous sinuses, and cannot be differentiated from the adenohypophysis itself. When suprasellar growth occurs, a characteristic “dumbbell” shape is characteristically seen, resembling a “snowman” appearance on coronal planes, with a central transverse constriction due to the presence of the diaphragm sella. The percentage of patients with cavernous sinus invasion at diagnosis is similar to the adult population, with about 85% showing a Knosp grade less than 2. On the other hand, large-size pediatric PitNETs are more likely to demonstrate internal heterogeneity due to cysts, hemorrhage and fluid-fluid levels (87) (Figure 5). Hemorrhage is even more common if bromocriptine therapy had been previously attempted (79, 88).

These large neuroendocrine tumors may be mistaken for other lesions, such as craniopharyngiomas or RCC. Differentiation between PitNETs and craniopharyngioma can be achieved when calcifications are present and can be improved by using more advanced imaging tools. Apparent diffusion coefficient (ADC) values, derived from turbo spin-echo DWI, are consistently lower in PitNETs than in craniopharyngiomas (89). MR spectroscopy can also be useful, showing either a choline peak or complete absence of metabolite peaks, while craniopharyngiomas demonstrate a lactate/lipid peak.

### 4.5. Pituitary blastoma

Pituitary blastoma, a rare embryonal sellar tumor of infancy, has been added as a pituitary tumor type in WHO CNS5. It harbors three distinct cellular components: undifferentiated blastemal cells, Rathke's pouch epithelium, and adenohypophyseal neuroendocrine cells, predominantly showing corticotroph cell differentiation (6, 72, 90).

Only a few dozens of pituitary blastomas have been published so far (6, 91). Individuals aged less than 2 years are typically affected, with a median age of 9 months and a slight female predominance. Nevertheless, these tumors are not restricted to childhood and may also occur in young adults (92, 93). Affected children present most frequently with elevated ACTH and Cushing disease, an exceedingly rare endocrinopathy in this age group (94). Occasionally, ophthalmoplegia, signs of increased intracranial pressure, diabetes insipidus, and thyrotropin deficit may be present.

Almost all published cases were heterozygous for a loss-of-function germline *DICER1* pathogenic variant. Indeed, pituitary blastoma is one of the hallmarks of *DICER1* syndrome, a highly pleiotropic genetic condition associated with increased risk of multiple hereditary tumors, both benign and malignant. This tumor predisposition syndrome is caused by variants in the homonymous gene (located at 14q32.13), that encodes an endoribonuclease playing a role in protein translational control, thereby affecting tumorigenesis. Approximately 20 hamartomatous, hyperplastic or neoplastic conditions comprise *DICER1* syndrome, including pleuropulmonary blastoma, multinodular goiter, cystic nephroma, and ovarian Sertoli–Leydig Cell and tumor nasal chondromesenchymal hamartoma (95). CNS and ophthalmological manifestations include metastasis of pleuropulmonary blastoma (96), as well as pineoblastoma (93), ciliary body medulloepithelioma (97), primary *DICER1*-associated CNS sarcoma (98) and embryonal tumor with multilayer rosettes-like infantile cerebellar embryonal tumor (99).

The neuroimaging appearance of pituitary blastomas is nonspecific and may vary from a small solid pituitary lesion to a large heterogeneous solid-cystic mass mimicking a macroadenoma (100). Nevertheless, presence of a sellar/suprasellar mass in a patient younger than 2 years should always raise the suspicion of a pituitary blastoma, especially if there is associated macrocrania (101) and family history of multiple tumors.

It remains uncertain if pituitary blastomas are low- or high-grade tumors and further evidence is needed on this topic. Although it is estimated that approximately 50% of affected children die of the disease (92), some studies suggest that most deaths are due to early or late treatment-related complications (102).

### 4.6. Langerhans cell histiocytosis and related disorders

Langerhans cell histiocytosis (LCH) and juvenile xanthogranuloma (JXG) are myeloid-derived clonal inflammatory disorders composed of dendritic or macrophage/monocyte derived cells. Both histiocytic tumors are listed in WHO CNS5 and can present as sellar/suprasellar masses (6).

LCH of the CNS is a clonal proliferation of Langerhans-type cells manifesting in the brain and/or spine, with or without associated systemic involvement. The meninges (30%) and choroid plexus (6%) can also harbor LCH tumors. On the other hand, craniofacial bone and skull base involvement occurs in 65% of cases and is the most common feature of LCH in the head and neck (6).

The most frequent subjacent molecular alteration in LCH is *BRAF* p.V600E mutation, which occurs in about 50% of cases (103).

The median age at diagnosis is 3.5 years and there is a slight male predominance, which however is not present under 1 year of age, the period when the incidence is higher (104).

**Figure 6:**
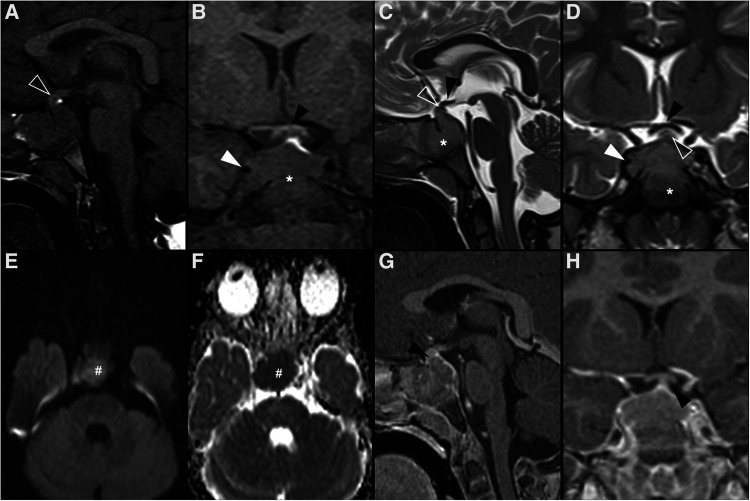
Langerhans cell histiocytosis in a 2-year-old girl presenting with right palpebral ptosis, headache and progressive diminished appetite and activity. Brain MR imaging reveals an expansile mass centered in the sphenoid body and sella turcica, homogeneously isointense to grey matter on coronal (**A**) and sagittal (**B**) T1WI, as well as and hyperintense on sagittal (**C**) and coronal (**D**) T2WI, mimicking a macroadenoma. The lesion presents suprasellar extension with infundibular thickening (empty white arrowheads), upward bowing of the central optic chiasm (white black arrowheads), as well as signs of clear invasion of the right cavernous sinus with carotid artery stenosis (black white arrowheads). One can clearly depict the preserved T1 hyperintensity of the compressed neurohypophysis in a left suprasellar position (white arrows). There is also destruction of the sellar floor invasion of the sphenoid bone and sinuses (asterisks). Note superior projection of the adenohypophysis and neurohypophysis (empty white arrowhead). Axial DWI (**E**) and ADC map (**F**) depict mild restricted diffusion suggestive of hypercellularity (hashtag). Contrast-enhanced sagittal (**G**) and coronal (**H**) T1WI reveal mild solid homogeneous enhancement of the lesion. Normal enhancement of the superiorly displaced adenohypophysis is present (black arrowhead). No other parenchymal, leptomeningeal or calvarial lesions were observed.

The most common intracranial manifestation is a mass involving the hypothalamic-pituitary axis (25%–50%), typically causing diabetes insipidus, that very rarely may be the sole clinical manifestation of the disease (Figure 6). The most frequent imaging features of hypothalamic-pituitary axis involvement in LCH are thickening [greater than 3 mm (105)] and enhancement of the pituitary stalk, associated with absence of the normal T1WI shortening of the posterior pituitary (106). As the pituitary stalk lacks blood-brain barrier, enhancement after gadolinium does not necessarily mean pathologic changes, which mandates a careful assessment of stalk thickness (12). The pituitary gland itself is affected in 10% of patients with CNS involvement (106). The second most common intracranial presentation of LCH consists of a neurodegenerative pattern with signal changes involving the dentate nuclei and the perinuclear cerebellar white matter, extending towards the pontine tegmentum and, occasionally, to the basal ganglia, with no mass effect or contrast enhancement (12, 106). Both basal ganglia and dentate nuclei frequently show T1WI hyperintense signal that may persist for several years (106, 107). These findings may precede a neurodegenerative syndrome with relentless progression that may also occur as many as 10 years after the presumed resolution of LCH mass lesions (108, 109). Diffuse CNS atrophy may develop over time (6). White matter changes may also appear, both in an asymptomatic perivascular spaces pattern (5%) or in a leukoencephalopathy-like pattern (36%) only found in severely disabled patients (106). Pineal cysts and enlargement of the pineal gland are prevalent findings in LCH patients, reflecting direct tumoral infiltration or hyperplasia. However, they lack specificity and can be found in other conditions where the hypothalamic-pituitary and pineal regions are simultaneously affected, like in the case of germinomas (106).

JXG is a histiocytosis characterized by foamy histiocytes, occasional Touton giant cells, and inflammation, overlapping its histopathologic features with Erdheim-Chester Disease (ECD). Importantly, it is the most common non-Langerhans cell histiocytic disorder in children. Most cases occur in the first 6 months of life and boys are slightly more affected than girls. JXG manifests as a cutaneous disease in up to 90% of cases, with a benign and self-limiting course. However, patients with systemic disease can have a severe clinical course, particularly when there is CNS involvement (1%–2.3%), which is extremely uncommon without cutaneous lesions. When extracutaneous disease is found, half of the patients may show isolated CNS involvement (110). In children, *BRAF* p.V600E mutation is not present in cutaneous-limited JXG, but only in cases with extracutaneous presentations, which suggests a related molecular pathogenesis with LCH and ECD. Together with similar clinical complications such as diabetes insipidus and/or neurodegenerative disease, these three conditions are now classified together in the “L” (Langerhans) group, according to the revised classification of histiocytes and neoplasms of the macrophage-dendritic cell lineages (111). Furthermore, detection of a *BRAF* p.V600E mutation in a JXG should prompt further work-up for pediatric ECD (112–114), and it is even recommended that all extracutaneous JXG with gain-of-function MAPK pathway mutations should be considered as ECD (111). The histologic differential diagnosis includes reactive xanthomatous processes and other histiocytic lesions, as they can be undistinguishable by imaging alone (112).

The majority of patients with CNS involvement show multiple intraparenchymal lesions, but unifocal and multifocal leptomeningeal patterns are also described as well as infundibular involvement (110, 112, 113). Intracranial xanthogranulomas have been mostly described as being T1- and T2- isointense comparing to cortical grey matter, although the presence of lipidized or xanthomatous cells may be responsible for T1- and T2 hyperintense signal and hypercellularity can translate into T2 hypointensity. Indeed, restricted diffusion is a common finding, as well as marked contrast enhancement (110, 115–117). In addition, and similarly to LCH, JXG family neoplasms may be accompanied by white matter neurodegeneration and atrophy (114).

However, although the sellar/suprasellar region is often affected in both JXG and LCH, intraparenchymal lesions are rather uncommon in the latter (106, 110).

The differential diagnosis on neuroimaging is not limited to other histiocytic disorders and also includes both tumoral entities, such as germ cell tumors, and autoimmune inflammatory conditions like lymphocytic hypophysitis (118).

Tumor-like LCH lesions are sensitive to conventional treatment and considered to be almost universally cured with chemotherapy (6, 107). In children with diabetes insipidus and a thickened pituitary stalk, after exclusion of a germ cell tumor (negative tumor markers) and lymphoma (negative CSF cytology), it may be reasonable to initiate empirical treatment, followed by MRI response monitoring (107). Bone lesions in the calvaria or skull base are “risk” lesions for CNS involvement, and should elicit special treatment considerations (108, 119). On the other hand, LCH neurodegenerative lesions are resistant or poorly sensitive to multiple therapeutic strategies (120, 121).

On the other side, and as stated above, intracranial JXG show a more aggressive behavior than the benign cutaneous JXG, with no published cases of spontaneous regression (122). Treatment varies considerably but most patients undergo surgery, while a smaller proportion receive chemotherapy and radiotherapy. LCH and JXG patients with *BRAF* p.V600E mutation in which the conventional treatment fails may benefit from newer targeted kinase inhibition (123, 124).

### 4.7. Germ cell tumors

Germ cell tumors (GCT) are a heterogeneous group of neoplasms derived from germ cells that can incorporate both early embryonic and mature elements. GCT account for 3%–4% of all pediatric brain tumors and have been classically considered more prevalent in eastern Asia than in Europe and the USA, although more recent studies challenge this concept (125). In addition, intracranial GCT are more frequently seen in males with Klinefelter syndrome and possibly in Down syndrome patients (126–128).

**Figure 7:**
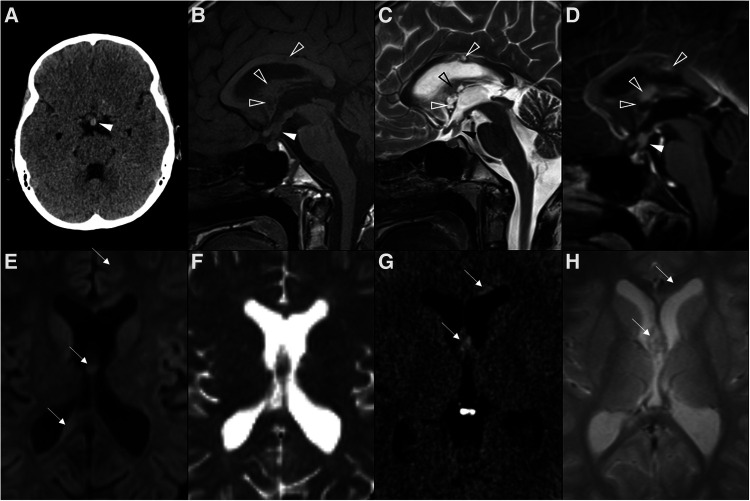
Multifocal germinoma in a 12-year-old boy with panhypopituitarism. Thickening of the pituitary stalk and tuber cinereum was observed in axial head CT (**A**) and sagittal T1WI (**B**) and T2WI (**C**), which enhanced homogenously after gadolinium in sagittal T1WI (**D**) (white arrowheads). The expected T1 hyperintensity of the neurohypophysis is absent. Also note additional solid-cystic lesions in the body of the corpus callosum, lamina terminalis, and ceiling of the third ventricle with associated contrast enhancement (empty white arrowheads), indicative of multifocal distribution. Despite no diffusion restriction was seen on DWI (**E**) and ADC map (**F**), all lesions were spontaneously hyperdense on CT (**G**) with no hypodense foci on T2 gradient-echo images (**H**), supporting some degree of hypercellularity (white arrows).

**Figure 8:**
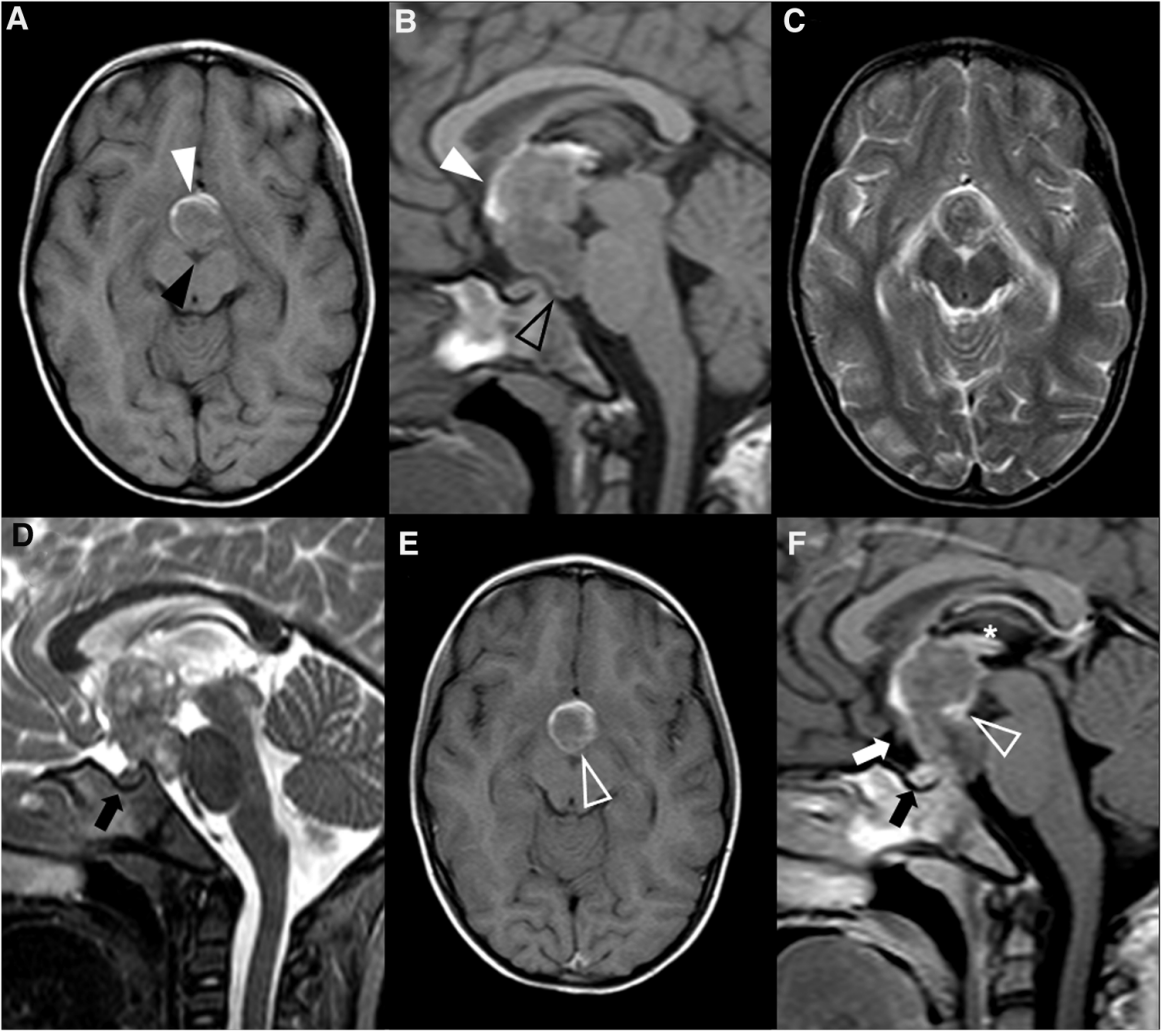
Teratoma in a 4-year-old girl. Brain MR imaging shows an expansile lesion separated from the pituitary gland, centered in the suprasellar cistern with extension into the interpeduncular (black arrowhead) and pre-pontine cisterns (empty black arrowhead). The lesion is mainly isointense on axial (**A**) and sagittal (**B**) T1WI, but presents a linear component spontaneously hyperintense anteriorly, that corresponds to adipose tissue (white arrowheads). On axial (**C**) and sagittal (**D**) T2WI the lesion shows heterogeneous signal. Post-contrast axial (**E**) and sagittal (**F**) T1WI shows only minor posterior peripheral enhancement (empty white arrowheads). There is moderate mass effect over the anterior recesses of the III ventricle (asterisk) and the optic chiasm (white arrow). Note normal positioning and enhancement of the adenohypophysis and absent hyperintense T1 bright spot of the neurohypophysis (black arrows).

**Figure 9:**
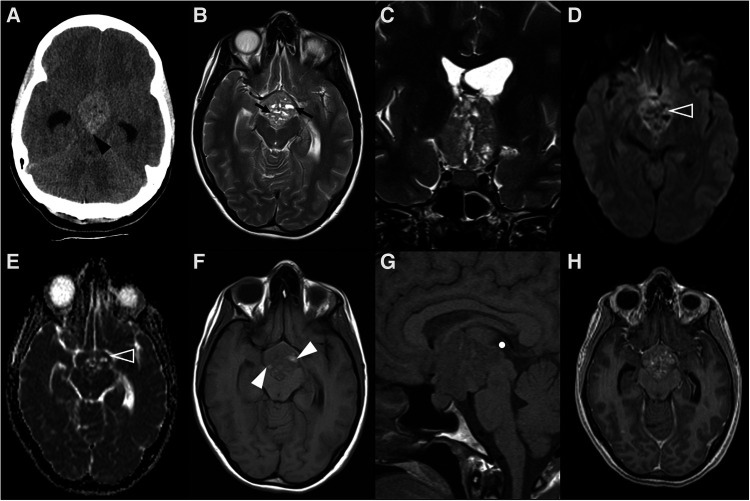
Choriocarcinoma in a 17-year-old female presenting with central diabetes insipidus. Axial head CT scan (**A**) reveals a spontaneously hyperdense mass centered in the supra-sellar cistern and with extension towards the interpeduncular cistern (black arrowhead). Brain MRI imaging including axial (**B**) and coronal (**C**) T2WI, DWI (**D**) and ADC map (**E**), as well as axial (**F**) and sagittal (**G**) T1WI, better depicts a polilobulated lesion, which is well demarcated from the pituitary gland. It shows heterogeneous signal, including levels (black arrows) and areas of spontaneous T1 hyperintensity (white arrowheads) in keeping with hemorrhagic/proteinaceous content, and foci of restricted diffusion (empty white arrowheads). The spontaneous T1 bright spot of the neurohypophysis cannot be detected on sagittal pre-gadolinium T1WI (**G**). On post-gadolinium axial, heterogenous enhancement is observed. Note the almost complete obliteration of the third ventricle (**C** and white circle in **G**), the optic chiasm and tracts (white arrows in **C**), and the cerebral peduncles (black arrowheads in **H**), as well as mild hydrocephalus (asterisks in **A,C**).

Molecular methods currently play only a minor role in the diagnosis and subclassification of GCT of the CNS, which generally rest on histopathological and immunophenotypic features (6). Germ cell tumors have been historically classified into germinomas (Figure 7) [that correspond to about 2/3 of intracranial GCT] and nongerminomatous [the latter including teratomas (Figure 8), yolk sac tumors, embryonal carcinomas and choriocarcinomas (Figure 9)] because of the distinct patterns of response to radiotherapy and chemotherapy (129). Pure germinomas outnumber other types, with mixed lesions and teratomas being the next most common (6).

Although GCT are predominantly localized in the gonads, 1%–5% are found in extra-gonadal localizations, usually along the midline of the body, due to the migration path of primordial germ cells during embryogenesis (128, 130). While the mediastinum and peritoneum are the main sites for adult extragonadal GCT, intracranial and sacrococcygeal regions are the most affected in children (131). In the CNS, they usually present in the suprasellar/infundibular region or in the pineal gland. Other possible locations include the basal ganglia and thalamus. Bifocal or multifocal presentation (usually corresponding to germinomas) may be detected in up to 15% of cases (4, 6, 132).

GCT can occur at all ages, but mainly affect children and young adults, with an incidence peak in patients aged 10–14 years. There is also an overall clear predilection for male patients, particularly when the pineal region is affected. However, there is no sex predilection in suprasellar cases (133).

Suprasellar GCT (more often corresponding to germinomas) usually manifest with diabetes insipidus, visual impairment, and hypothalamic-pituitary failure (6, 134). Importantly, germinoma is the most common tumoral cause of hypothalamic syndrome in children (134).

The MRI appearance of suprasellar GCT spans from a small homogeneous stalk or infundibular lesion with absence of the normal T1WI neurohypophyseal bright spot to a larger predominantly solid mass with avid contrast enhancement and signs of reduced free water content, namely iso- to hypointense T2 signal and restricted diffusion (also high density on CT) (12, 135). The former presentation overlaps with that of LCH, although a GCT diagnosis may be favored in the presence of hypothalamus infiltration or unilateral basal ganglia involvement, atrophy and Wallerian degeneration (135, 136). A recent study by Esfahani et al. (137) reviewed clinical and radiographic features of 42 pediatric suprasellar GCTs and found notable morphologic differences between solitary GCT and bifocal GCT. Bifocal GCT tended to have small suprasellar components (usually nodular or laminar on the floor of the III ventricle and infundibulum) and much larger pineal region tumors, while almost all solitary GCT were globular masses presumably originating from the III ventricle floor, the majority of these extending rostrally and forming a prominent anterior III ventricle lesion. Less commonly, solitary GCT can extend downwards the sella turcica without a III ventricle mass. The authors proposed a MR-based classification system regarding suprasellar GCT. In addition, bifocal GCT presented at an older age, were also more frequent in boys and more often associated with ventricular metastasis. Importantly, CNS GCT have the propensity for invasion and CSF dissemination, thus the entire neuraxis should be imaged in suspected cases (129).

The distinction between germinomas and nongerminomatous GCTs is important, as the extent of surgery, radiation field, and chemotherapy regimens differ between the two major GCT subgroups, as it will be addressed below (138). Neuroimaging, including DWI/ADC maps, can also help in this differentiation as both ADC_mean_ and ADC_max_ values are notably lower in germinoma than in the other subtypes (138). Furthermore, suprasellar germinomas present less frequently with intratumoral hemorrhage when compared with the other subtypes, explaining the lack of T1 hyperintense and T2*/SWI hypointense foci on pre-treatment MRI (138, 139). Conversely, the majority of basal ganglia germinomas show low T2*/SWI signal due to hemorrhagic foci (139).

Measurement of oncoproteins in the serum or CSF in suspected patients can be of extreme relevance. By definition, pure germinomas do not secrete beta-human chorionic gonadotropin (β-HCG), although mild elevations are seen in the syncytiotrophoblastic form of germinomas. Nongerminomatous GCT can secrete both β-HCG and α-fetoprotein, the latter being more specific for yolk sac tumors (140).

As germinomas are highly sensitive to both chemotherapy and radiotherapy, surgery is usually limited to biopsy, urgent decompression, and/or excision of residual tumor after chemo- and radiotherapy treatment. Most nongerminatous GCTs are also chemo- and radiosensitive, although to a lesser extent. On the other hand, surgery remains the only curative option in mature teratomas (129, 141).

The prognosis of GCT varies by histology. Mature teratomas and pure germinomas share the best outcomes, exceeding 90% survival rates over 10 years of follow-up. On the other hand, immature (usually congenital) teratomas, intracranial yolk sac tumors, embryonal carcinomas, and choriocarcinomas in pure form are characterized by dismal outcomes (142). Intracranial GCT mostly recur within the first 20 months after the initial treatment. Invasiveness at presentation and the presence of cysts were found to be frequent among patients with recurrence (143). Interestingly, the number of lesions (single, multifocal, or bifocal) seems not to have an important impact on overall survival or relapse-free survival (138, 144), although there is a trend to worse prognosis in bifocal GCT (137). Moreover, there is a general consensus that bifocal germinomas can be safely treated as localized tumors rather than metastatic disease (141). Finally, long-term survivors of both germinoma and nongerminomatous tumors remain at risk of premature death due to treatment-associated effects (145).

### 4.8. Hypothalamic and optic pathway astrocytoma

Although hypothalamic and optic pathway astrocytoma (or simply optic pathway glioma—OPG) is not a formal entry in the new WHO CNS5, it is worth a mention since it represents 25 to 30% of suprasellar neoplasms and 4% of all intracranial tumors in pediatric population (13). This nomenclature derives from the fact that gliomas originating in the optic chiasm or in the hypothalamus often grow to involve both structures, with its primary origin site being usually undetermined (10). More than half of these tumors are diagnosed in the first 5 years of life and there is no gender predilection (146, 147).

**Figure 10:**
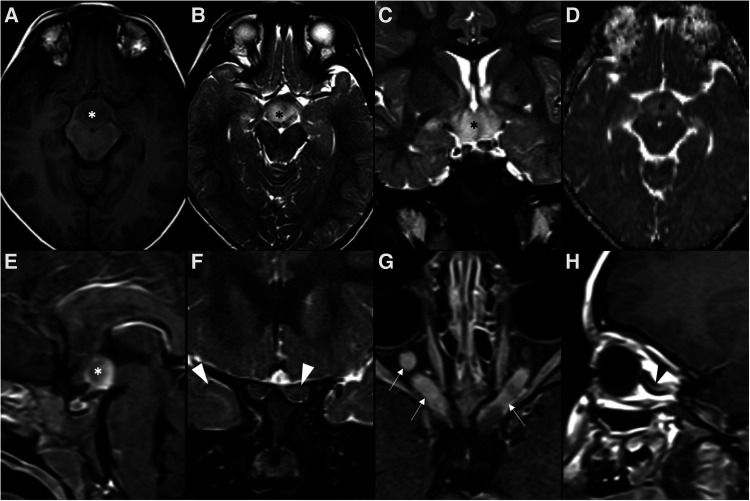
Neurofibromatosis type 1-related hypothalamic and optic pathway astrocytoma in a 3-year-old boy. Brain MRI axial T1WI (**A**), and axial (**B**) and coronal (**C**) T2WI show a T1 hypointense and T2 hyperintense suprasellar infiltrative neoplasm (asterisks), with facilitated diffusion on ADC map (**D**) and solid enhancement on sagittal T1WI after paramagnetic contrast media injection (**E**). Coronal T2W images with fat suppression (**E**) depict anterior extension to both optic nerves, with T2 signal change and increased thickness (white arrowheads). Enhancement (white arrows) and downward kinking (black arrowhead) of the orbital segment of the optic nerves can be seen on axial (**G**) and left paramedian sagittal (**H**) T1WI, respectively. Several focal areas of signal intensity (FASI), including on the left globus pallidus (black arrow on **C**), were detected.

**Figure 11:**
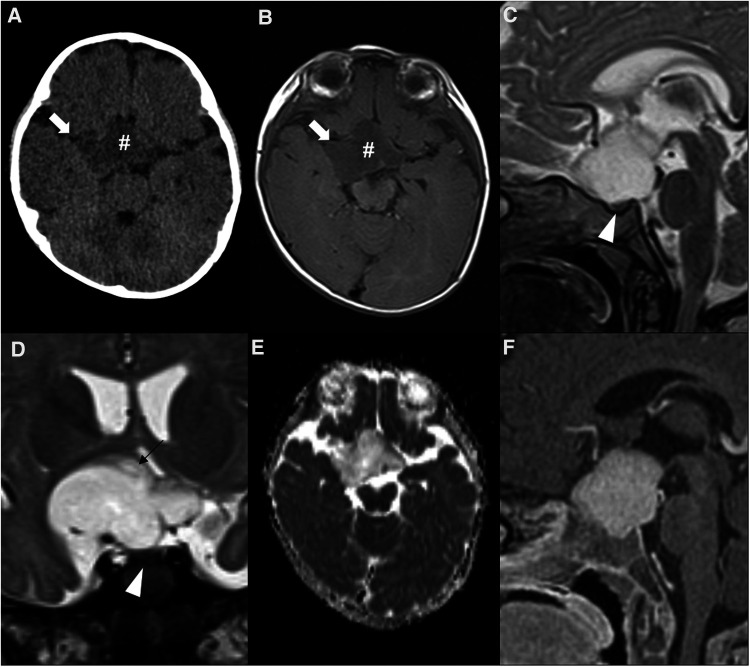
Sporadic hypothalamic and optic pathway astrocytoma in a 7-month-old girl presenting with horizontal nystagmus. Axial head CT (**A**) and axial brain MRI T1WI (**B**) shows a hypodense and hypointense expansile lesion (hashtags) centered in the suprasellar region, with extension towards the sylvian fissures, mainly on the right (white arrows). Sagittal (**C**) and coronal (**D**) T2WI show a T2 hyperintense lesion which is separated from the pituitary gland (white arrowheads) and originating posterior from the optic chiasm, most probably from the right hypothalamus (black arrow in **D**). The chiasm and optic tracts (right optic tract is pointed out by the black arrowhead in **D**) are splayed but not overtly infiltrated. There are no optic nerve changes (not shown). Facilitated diffusion is seen on ADC map (**E**), as well as homogeneous contrast enhancement on sagittal T1WI after gadolinium injection (**F**).

Twenty to 50% of OPGs occur in the setting of neurofibromatosis type 1 (NF1) (Figure 10) and, in turn, 15% of NF1 patients will develop an OPG (13, 148). Almost all of these tumors are grade I pilocytic astrocytomas, the majority being located within the optic nerve and chiasm (149). The remaining cases are sporadic (Figure 11) and more often involve the posterior optic pathways and hypothalamus, showing a slightly more aggressive radiological and clinical behavior, despite being histologically similar to NF1-associated OPG (150). On the other hand, the pilomyxoid astrocytoma is another subtype of astrocytoma also characteristically occurring in the hypothalamic/chiasmatic region in the pediatric age, with a higher rate of recurrence and a poorer outcome than classic pilocytic astrocytoma, probably due to a higher propensity for cerebrospinal dissemination (151). Despite their differences, both these tumors are associated with genetic abnormalities in genes encoding members of the MAPK pathway, most commonly resulting in *BRAF* gene abnormalities (6).

Clinical manifestations of OPGs include slowly progressive visual impairment, with or without other ophthalmologic complaints (more common in NF1-associated OPG) and optic atrophy. On the other hand, endocrinological changes are seen in about 20% of cases while hypothalamic impairment and hydrocephalus can also occur in a small subset of patients, more commonly in sporadic OPG due to their more posterior location. Moreover, OPG may also cause the Russelĺs diencephalic syndrome in small children, which is characterized by progressive emaciation and failure to thrive in an apparently alert infant (4, 12, 152).

Hypothalamic and optic pathway astrocytomas are usually well marginated tumors, with multilobular, oval or rounded shape. They tend to be hypointense on T1WI and hyperintense on T2WI, with increased T2 heterogeneity in larger masses (12) while DWI typically shows increased water diffusivity in keeping with the characteristic low cellularity of these tumors. Contrast enhancement is typically present and heterogeneous, often in the peripheral portion of the lesion, but may be mild or even absent. The pattern of enhancement may also change throughout the clinical course, including under chemotherapy. In fact, an increase in enhancement without an increase in size should not be interpreted as progression (153, 154).

In the precise setting of NF1, lesions in the optic tract/hypothalamus with T2 hyperintensity should be defined as probable tumor if associated with T1 hypointensity relative to white matter, and/or show mass effect or enhancement (154). In addition, a few morphologic features like smaller size, tumor shape stability, predominantly solid composition/absence of cystic components, and downward kinking of the retrobulbar optic nerves characterize NF1-related OPG comparing to sporadic ones (10, 12, 155).

Moreover, a recent study found measurable differences between NF1-related OPG, sporadic OPG and healthy controls in the subventricular zone of the third ventricle, both in terms of ADC and cerebral blood flow (CBF) quantification, with NF1-related tumors showing higher ADC values and lower CBF comparing to the other groups (150).

On the other hand, presence of intralesional hemorrhage should raise suspicion for pilomyxoid astrocytoma and therefore mandates a thorough evaluation of CSF seeding (12).

Long-term management of patients with OPG is based upon serial MRI studies, along with clinical and ophthalmological evaluations. Interestingly, pilocytic astrocytomas may even involute spontaneously, wether or not in the setting of NF1. On the other hand, treatment is generally initiated only when radiological progression and/or clinical deterioration are found (156) and usually includes surgical removal and/or chemotherapy and radiation therapy. However, because of the favorable overall outcome, radiation-sparing approaches are commonly recommended, with chemotherapy being advocated as the initial treatment strategy (157). In addition, as most of these tumors harbor alterations in MAPK pathway genes, targeted MEK inhibition has also been attempted with good results (6).

An anatomical classification for OPG was proposed by Dodge et al. in 1958 (158) and has since then been used to select patients for resection of optic nerve tumors. Other classifications were published more recently, which can better help on treatment decision-making (152, 159).

Finally, care must be taken when interpreting total tumor volume changes in patients previously treated with proton beam therapy or immunotherapy, since considerable cystic changes and paradoxical imaging findings can occur (160, 161).

### 4.9. Suprasellar atypical teratoid/rhabdoid tumor

Atypical teratoid/rhabdoid tumor (AT/RT) is an aggressive high-grade (CNS WHO grade 4) malignant neoplasm containing poorly differentiated cells and a variable number of rhabdoid cells. In the vast majority of cases, the genetic hallmark of these tumors is a pathogenic variant in the *SMARCB1* tumor suppressor gene located on chromosome 22q, that codes for a subunit (INI1) of the SWI/SNF chromatin remodeling complex. In less than 5% of cases, biallelic inactivation of *SMARCA4* occurs instead (162, 163).

Initially reported in the kidney, the first apparent case of a primary CNS rhabdoid tumor was reported in 1987 and since then multiple cases have been published (164). AT/RT is thought to represent approximately 3% of all pediatric CNS tumors, about 10% in children aged less than 1 year, and up to 20% of CNS tumors in children under the age of 3 years (165). Published data is conflicting regarding the most common location for AT/RT (165–167). Nevertheless, sellar/suprasellar AT/RT is thought to be rare, with very few cases overall reported. More specifically, only 2 cases were identified among 143 pediatric patients with AT/RT from 13 European countries (167).

Three molecular groups with different methylation and transcriptional signatures have been designated by consensus, namely as AT/RT-TYR, AT/RT-SHH, and AT/RT-MYC, showing differences in patient age, localization, and *SMARCB1*/chromosome 22 alteration patterns (6). Interestingly, adult sellar AT/RT belongs to the AT/RT-MYC group, while no unique group was linked so far to pediatric sellar AT/RT (168).

In the suprasellar region, AT/RT can present with a wide variety of signs and symptoms, including headache, diplopia, subarachnoid hemorrhage, diabetes insipidus, and/or panhypopituitarism (169).

On imaging, regardless of the CNS location, these tumors frequently present as large heterogeneous masses with solid, cystic and necrotic components. On CT, hyperdense areas due to hypercellularity, calcifications (50%) and/or hemorrhage can be seen, and skull invasion has been reported (170). Because of their high cellularity, MRI characteristics are similar to those of medulloblastomas, although eccentrically located cysts and rapid growth favor the diagnosis of AT/RT in other intracranial locations and may also be a clue in sellar/suprasellar masses despite the scarcity of data (170, 171). In particular, although the signal intensity of these tumors is variable both on T1WI and T2WI, diffusion restriction is typical, reflecting densely packed cellular arrangement. In addition, more than half of the cases reveal contrast enhancement in more than two thirds of the tumor volume. Proton MR spectroscopy is nonspecific, usually showing elevated levels of choline and decreased N-acetylaspartate (171). Importantly, AT/RTs have a propensity for seeding the CSF pathways, which can be seen in one third of cases and entire neuroaxis imaging should be performed in suspected cases (172).

The overall prognosis of patients with AT/RT is poor, with presence of disseminated leptomeningeal tumor at diagnosis, age less than 1 year and “non-TYR” molecular subgroups being associated with worst outcomes (167, 171). However, data from retrospective studies and clinical trials have shown that some subgroups of patients with AT/RT do not always have a dismal outcome when multimodal treatment strategies (surgery, chemotherapy, radiation) are employed (6, 167, 173, 174). The different epigenomic configurations of AT/RT subtypes could be associated with distinct vulnerabilities, including immune checkpoint inhibition as a potential therapeutic strategy (6).

## 5. Discussion

The 5th edition of the WHO classification of CNS neoplasms incorporated both histologic and molecular alterations into a common diagnostic framework, with impact in the classification and grading of sellar/suprasellar tumors. Since the first developmental steps, this anatomical region is one of the most complex places of the intracranial compartment, where endocrine, nervous, vascular and CSF-filled structures tightly converge, leading to an extraordinary variety of neoplasms. Moreover, childhood tumors are fundamentally different from those occurring in adults and raise distinctive diagnostic, therapeutic and prognostic considerations. Although the current molecular landscape is the fundamental driving force to the new WHO CNS tumor classification, the imaging profile of sellar/suprasellar tumors remains largely unexplored, particularly in the pediatric population. Moreover, after clinical assessment, neuroimaging still offers the first complete information about sellar/suprasellar mass lesions in children, and can provide with invaluable diagnostic and prognostic information.

**Figure 12:**
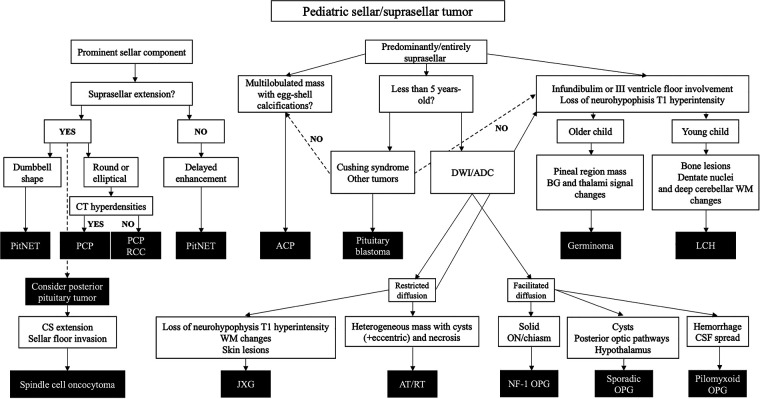
Imaging-based diagnostic algorithm for pediatric sellar/suprasellar tumors. ADC, apparent diffusion coefficient; APC, adamantinomatous craniopharyngioma; AT/RT, atypical teratoid/rhabdoid tumor; BG, basal ganglia; CS, cavernous sinus; CT, computed tomography; DWI, diffusion weighted imaging; GCT, germ cell tumor; JGX, juvenile xanthogranuloma; LCH, Langerhans cell histiocytosis; NF-1, neurofibromatosis type 1; ON, optic nerve; OPG, optic pathway glioma; PCP, papillary craniopharyngioma; PitNET, pituitary neuroendocrine tumor; RCC, Rathke’s cleft cyst; WM, white matter.

We have reviewed the radiological features of all neoplasms listed as “tumors of the sellar region” in the WHO CNS5, and further added glial, embryonal, histiocytic and germ cell tumors with variable preferences to sellar/suprasellar involvement. Based on the research performed and on the author's experience, we propose an imaging-based diagnostic algorithm to aid in individual cases diagnostic workup (Figure 12).

ACP and PCP are now known to be different neoplasms since they have distinct methylation and transcriptional profiles. Pediatric PCP—a rare disease—differs from adult counterparts by consistently showing prominent cystic components and a subdiaphragmatic origin. Bifocal mass lesions involving the pineal and suprasellar regions correspond almost invariably to germinomas. Absence of the normal T1WI neurohypophyseal bright spot is typical of LCH although suprasellar GCTs can present in the same way, with basal ganglia or hypothalamus infiltration favoring the diagnosis of GCT. Distinguishing germinomas and nongerminomatous GCTs may be achieved with the assessment of ADC values and intratumoral hemorrhage. Particular neuroimaging features like the location within the optic pathway, the tumor volume, the presence of hemorrhage and signs of CSF dissemination, may help on the prediction of OPG subtypes. Despite being extremely rare tumors, the presence of a sellar/suprasellar mass in small children should raise the suspicion of a pituitary blastoma or an AT/RT.
