# Supplementary material for: Neuroimaging of pediatric tumors of the sellar region—A review in light of the 2021 WHO classification of tumors of the central nervous system
Source: Front Pediatr. 2023 Jun 21;11:1162654. doi: 10.3389/fped.2023.1162654 (PMC10320298; doi:10.3389/fped.2023.1162654)
Supplement: Supplementary file 1 [file Datasheet1.pdf]

Supplementary Material

Neuroimaging of Pediatric Tumors of the Sellar Region

Rúben Maia[1], André Miranda[2,3], Ana Filipa Geraldo*[2,4], Luísa Sampaio[1,5], Antonia Ramaglia[6], Domenico Tortora[6], Mariasavina Severino[6], Andrea Rossi[6,7].

[1] Department of Neuroradiology, Centro Hospitalar Universitário São João, Porto, Portugal

[2] Diagnostic Neuroradiology Unit, Imaging Department, Centro Hospitalar Vila Nova de Gaia/Espinho, Vila Nova de Gaia, Portugal

[3] Life and Health Sciences Research Institute (ICVS), School of Medicine, University of Minho, Campus Gualtar, Braga, Portugal

[4] Faculty of Medicine, University of Lisbon, Lisbon, Portugal

[5] Faculty of Medicine, University of Porto, Porto, Portugal

[6] Neuroradiology Unit, IRCCS Istituto Giannina Gaslini, Genoa, Italy

[7] Department of Health Sciences (DISSAL), University of Genoa, Genoa, Italy

*** Correspondence:**
Corresponding Author
anafilipageraldo@gmail.com

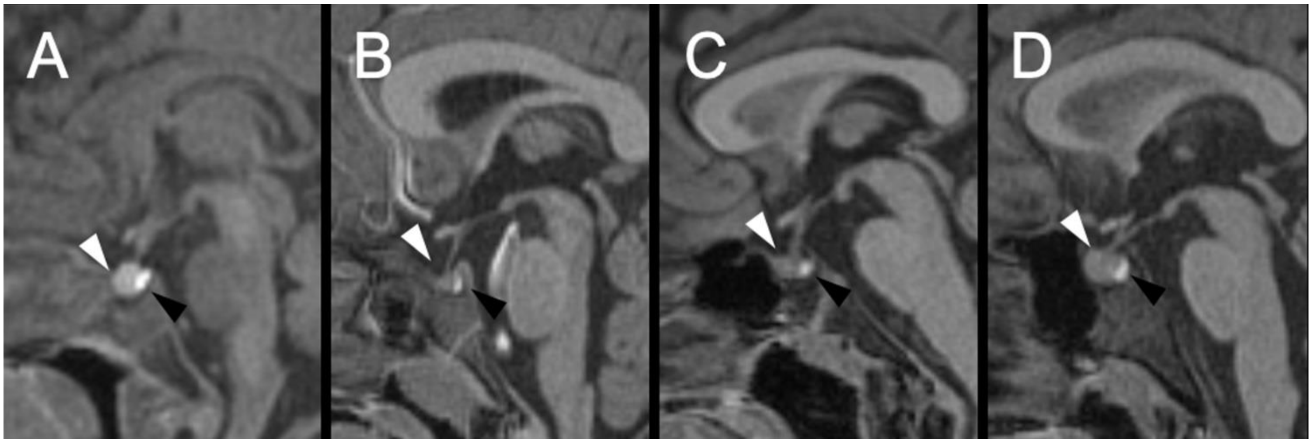

Supplementary Figure 1. Physiological changes of the pituitary gland according to age and/or gender. Sagittal T1WI in a 9-day-old female term neonate (A) shows a large pituitary gland, with age-appropriate T1 hyperintensity in both the adeno- and neurohypophysis. Sagittal T1WI in a 4-year-old boy (B) and a 14-year-old boy (C) reveal normal positioning of the pituitary gland in the sella turcica, with isointense adenohypophysis and expected superior concave borders (white arrowhead) as well as adequate preservation of spontaneous T1 hyperintensity of the neurohypophysis (black arrowhead). Sagittal T1WI in a 16-yearl-old girl (D) demonstrates physiological hyperplasia of the adenohypophysis, with superior convex borders resembling a spherical shape (asterisk).

Supplementary Table 1. Summary of the main features of pediatric sellar and suprasellar tumors.

	ACP	PCP	PPT	PitNET	PB	LCH	JXG	GCT	OPG	AT/RT
**Molecular/ Laboratory /Genetic**	CTNNB1 (activating)	BRAF p.V600E	TTF1	95% sporadic<br>5% familial<br><br>Usually ↑ Prolactin or ACTH	DICER1	BRAF p.V600E (50%)	BRAF p.V600E (only in extracutaneous disease)	↑ α-fetoprotein and/or<br>↑ β-HCG	BRAF	SMARCB1 (95%)<br>SMARCA4 (5%)
**WHO grade**	1	1	Low grade	-	Controversial	-	-	-	PA: 1<br>PmA: 2	4
**Frequency**	Almost all CP in children	Rare, especially in children	Rare, especially in children	Uncommon in children	Rare, but almost all cases presenting in children	CNS involvement: 25%	Rare	3-4% of all pediatric brain tumors	4% of all pediatric brain tumors	20% occur in children <3Y, but rare in the suprasellar region
**Gender**	M=F	M>F	-	Pre-Puberty: M=F<br>Post-Puberty: F>M<br>Prolactinoma: F>>M	F>M	<1 Y: M=F<br>>1 Y: M>F	M>F	*Overall:* M>>F<br>*Suprasellar:* M=F<br>Bifocal: M>F	M=F	-
**Age predilection**	5-14 Y	Adults<br>Adolescents (17-18 Y)	Adults	Adults<br>Adolescents	< 2 Y	Median: 3.5 Y	< 6 months	10-14 Y<br>Bifocal: older patients	<5 Y	<3 A
**Main location within sellar/suprasellar region**	Suprasellar	Sellar + suprasselar, but subdiaphragmatic	Sellar + suprasellar	Sellar ± suprasellar	Sellar and suprasellar	Pituitary stalk<br>Sphenoid	Pituitary stalk	Suprasellar<br>Pituitary Stalk	NF-1: ON and chiasm<br><br>Sporadic: posterior optic pathway and hypothalamus	Suprasellar
**Sellar/suprasellar tumor morphologic features**	90% Cysts<br><br>90% Calcifications	Cysts<br><br>Hiperdense components on CT		Lesions not separated from the pituitary gland<br><br>Deviation of the pituitary stalk<br><br>If large: “snowman appearance”; cysts and Intratumoral hemorrhage may occur	Nonspecific	Pituitary stalk thickening and enhancement<br><br>Absence of the neurohypophysis T1WI shortening	Pituitary stalk thickening and enhancement<br><br>Absence of the neurohypophysis T1WI shortening	Pituitary stalk thickening and enhancement<br><br>Absence of the neurohypophysis T1WI shortening<br><br>NGGCT tumors: intratumoral hemorrhage more common<br><br>Teratomas: fat tissue and/or calcification / bone / teeth	NF-1 PA: smaller and solid lesions<br><br>Sporadic PA: larger lesions, often with cysts<br><br>PmA: intratumoral hemorrhage	Solid, hemorrhagic and cystic (++ eccentric) components<br><br>Hyperdense on CT<br><br>Calcifications
**Contrast enhancement**	+ Solid component and cyst walls	+ Cyst walls	+	Delayed enhancement on DCEI	+	+	+	+	-/+<br>peripheral	+<br>heterogeneous

**Advanced imgaging**	H-MRS: ↑ Lipid peak			H-MRS: ↑ Cho peak or absent metabolites		Restricted diffusion	Restricted diffusion	Restricted diffusion (ADC lower in germinomas than in NGGCT)	Facilitated diffusion<br><br>NF1 PA: ↑ADC and ↓CBF in the subventricular zone	Facilitated diffusion<br><br>H-MRS: ↑Cho; ↓NAA
**Meningeal involvement**	-	-	-	-	-	Leptomeningeal lesions may occur (30%)	Leptomeningeal lesions may occur	CSF spread	PmA: CSF spread	CSF spread
**Other features**	-	-	Hypervascular tumor<br>Risk of surgical bleeding	ACTH-producing micro: 3D T1 GE is superior to DCEI	Suspect if <2Y with macrocrania and/or family history of multiple tumors	Bone lesions<br><br>Dentate nuclei/basal ganglia/WM degenerative signal abnormalities<br><br>Systemic masses	Cutaneous lesions<br><br>Unifocal or multifocal intracranial xanthogranulomas<br><br>WM degeneration and atrophy	Focal or multifocal intracranial lesions	NF-1 stigmata (20-50%):<br>e.g:<br>FASI<br>Neurofibroma<br>Lisch nodules<br>*Café-au-lait* spots<br>...	-
**Associated syndromes**	-	-	-	MEN-1<br>Carney's complex<br>McCune-Albright syndrome<br>Nelson syndrome	DICER1 syndrome	-	Erdheim-Chester disease	Klinefelter syndrome<br>Down syndrome	NF1 (20-50%)	RTPS

***Abbreviations**: ACP - Adamantinomatous craniopharyngioma; ACTH – Adrenocorticotropic hormone; ADC – Apparent diffusion coefficient; ATRT - Atypical teratoid/rhabdoid tumor;* β-HCG – Beta-human chorionic gonadotropin; CBF – Cerebral blood flow; *Cho – Choline; CNS – Central nervous system; CP – Craniopharyngioma; CSF – Cerebrospinal fluid; CT – Computed tomography; DCEI – Dynamic contrast-enhanced imaging; F – Females; FASI – Focal areas of signal intensity; GCT- Germ cell tumors; GE – Gradient echo; H-MRS – Proton MR spectroscopy; JXG- Juvenile xanthogranuloma; LCH - Langerhans cell histiocytosis; M – Males; MEN – Multiple endocrine neoplasia, type 1; NF-1 – Neurofibromatosis type 1; NGGCT – Non-germinatous germ cell tumor; ON – Optic nerve; OPG – Optic pathway glioma; PA – Pilocytic astrocytoma; PB - Pituitary blastoma ; PCP – Papillary craniopharyngioma; PitNET - Pituitary neuroendocrine tumor; PmA – Pilomyxoid astrocytomas; PPT – Posterior pituitary tumor; RTPS – Rhabdoid tumor predisposition syndrome; WM, White matter; WHO – World Health Organization; Y – Years*
